# Anthony Hale BSc (Psychology), BA (Archaeology), PhD, FRCPsych

**DOI:** 10.1192/bjb.2025.18

**Published:** 2025-12

**Authors:** Cornelius Katona, Marios Adamou

Formerly Emeritus Professor of Psychiatry, University of Kent, UK

**Figure:**
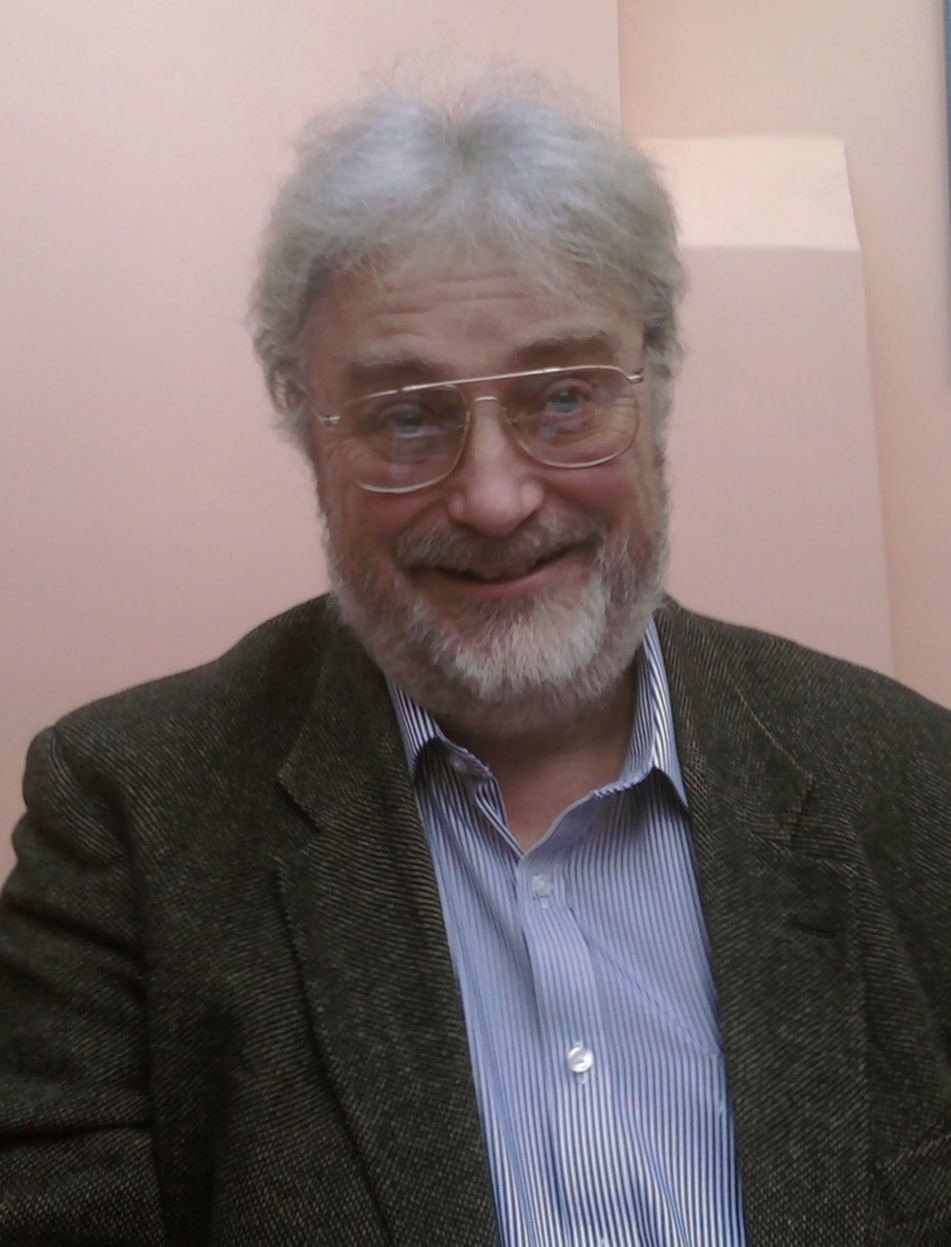

Professor Anthony (Tony) Hale, a psychiatrist, academic and educator of exceptional calibre, whose career was defined by scientific rigour, compassionate patient care and a profound commitment to teaching, died on 23 March 2024 after a prolonged illness. His life was shaped by an unrelenting intellectual curiosity and an uncompromising pursuit of excellence, placing him among the leading psychiatrists of his generation. His academic contributions addressed subjects as diverse as psychopharmacology, comorbidity and the legal complexities of psychiatric injury. His co-authored book *Neurology for Psychiatrists* remains a valuable resource in psychiatric education.

Tony was born in Portsmouth on 6 March 1955. His father, Alan Hale, was a mechanical engineer and technical author, and his mother, Jean Hale (née Wallis), was a hairdresser. He spent his childhood in the Portsmouth, Rochester and Cheltenham areas. His early years were marked by adversity. His mother’s sudden death when he was seven led to a period of upheaval, during which he and his younger siblings were separated until their father remarried. In adolescence and early adulthood, Tony regarded his intellect as his greatest asset, finding solace in learning and creativity.

He studied medicine at Guy’s Hospital, where he achieved an intercalated BSc in psychology with first-class honours. Although deeply engaged with experimental psychology, his fascination with the complexity and breadth of psychiatry led him to pursue medical training in the discipline. During his undergraduate years, he met Denise (née Greaves), a psychology student, whom he married in 1977. Together, they had a daughter, Bess, whose presence was a source of immense joy.

Tony trained in psychiatry at Guy’s under the mentorship of Professor Jim Watson, whose unwavering commitment to scientific precision profoundly influenced him. His early research focused on outcomes in psychosurgery before progressing to clinical trials with Professor Eugene Paykel at St George’s Hospital. Appointed senior lecturer and National Health Service (NHS) consultant at St Thomas’ Hospital in 1987, Tony’s broad intellectual interests flourished. His work in liaison psychiatry and psychopharmacology, including a secondment with Eli Lilly and Company, laid the foundation for his later research on antidepressants, antipsychotics and psychostimulants, culminating in a PhD in 1994.

In 1995, he was appointed professor of psychiatry at the University of Sheffield. However, this period proved personally challenging, as he experienced episodes of recurrent depression, a condition that would trouble him throughout his later life but also inform his teaching and his research. In 1997, with the support of his friend and former colleague Dr Steve Wood, he moved to a chair at the University of Kent, where he also served as a consultant psychiatrist within the local mental health trust. His dedication to patient care remained unwavering, particularly in the psychiatric intensive care unit, where he continued practising until his NHS retirement in 2013. He was granted emeritus professor status by the University of Kent.

Tony was an exacting and principled clinician, known for his uncompromising standard of care, which included meticulous consultations and detailed clinical correspondence. He appreciated the irony of having a condition within his own area of academic expertise and sometimes chose to share his experiences of depression with patients, recognising that this openness could help them feel less isolated. Long before the formalisation of the duty of candour, he was a staunch advocate for transparency in medicine. His high standards sometimes led to tensions with senior colleagues, particularly when he felt that clinical or academic integrity was at stake.

As an educator, Tony was profoundly committed to his students, refusing to oversimplify complex topics and challenging them to engage with the intellectual depth of psychiatry. His influence extended beyond the lecture hall, inspiring future psychiatrists and encouraging junior colleagues to collaborate on research and writing.

Tony’s intellectual pursuits extended beyond psychiatry. A resolute and knowledgeable amateur historian, he completed a BA in archaeology with first-class honours at the University of Kent in 2012 while formulating a whodunit theory regarding an ancient mystery, which he had hoped to explore in a novel. His characteristic wit and creativity were evident in the gentle cartoons he would sketch to enliven lectures and meetings.

At 58, Tony transitioned from the NHS to roles in the pharmaceutical industry and the independent sector, including work in asylum assessments. However, his final years were overshadowed by treatment-resistant depression and cognitive decline, later diagnosed as Parkinson’s disease with Lewy body dementia. His insight into his condition remained preserved, making this period particularly difficult for him and his family.

Tony is survived by Denise, Bess and a profound professional legacy. His influence on psychiatry – through rigorous research, dedicated patient care and an indelible impact on medical education – secures his place among the most respected psychiatrists of his time. His success despite his disability serves as a model for us as psychiatrists and for our patients.

